# The Effect of Animation-Guided Mindfulness Meditation on the Promotion of Creativity, Flow and Affect

**DOI:** 10.3389/fpsyg.2022.894337

**Published:** 2022-05-26

**Authors:** Hao Chen, Chao Liu, Fang Zhou, Chao-Hung Chiang, Yi-Lang Chen, Kan Wu, Ding-Hau Huang, Chia-Yih Liu, Wen-Ko Chiou

**Affiliations:** ^1^School of Film Television and Communication, Xiamen University of Technology, Xiamen, China; ^2^Business Analytics Research Center, Chang Gung University, Taoyuan City, Taiwan; ^3^School of Journalism and Communication, Hua Qiao University, Xiamen, China; ^4^Department of Economic and Management, Suzhou Vocational Institute of Industrial Technology, Suzhou, China; ^5^Department of Shipping and Transportation Management, National Penghu University of Science and Technology, Magong, Taiwan; ^6^Department of Industrial Engineering and Management, Ming Chi University of Technology, New Taipei, Taiwan; ^7^Department of Orthopaedic Surgery, Chang Gung Memorial Hospital, Taoyuan City, Taiwan; ^8^Institute of Creative Design and Management, National Taipei University of Business, Taoyuan City, Taiwan; ^9^Department of Psychiatry, Chang Gung Memorial Hospital, Taoyuan, Taiwan; ^10^Department of Industrial Design, Chang Gung University, Taoyuan City, Taiwan

**Keywords:** animation guided meditation, mindfulness, creativity, flow, positive affect

## Abstract

Creativity is so important for social and technological development that people are eager to find an easy way to enhance it. Previous studies have shown that mindfulness has significant effects on positive affect (PA), working memory capacity, cognitive flexibility and many other aspects, which are the key to promoting creativity. However, there are few studies on the relationship between mindfulness and creativity. The mechanism between mindfulness and creativity is still uncertain. Meditation is an important method of mindfulness training, but for most people who do not have the basic training, it’s difficult to master how to get into a state of mindfulness. Animation has been shown by many studies to help improve cognition and is often used as a guiding tool. Using animation as the guiding carrier of meditation is more convenient and easier to accept. Therefore, this study adopted the intervention method of animation-guided meditation, aiming to explore: (1) the effect of animation-guided meditation on enhancing creativity; (2) the role of flow and emotion in the influence of mindfulness on creativity. We advertised recruitment through the internal network of a creative industrial park, and the final 95 eligible participants were divided into two groups: animation (*n* = 48) and audio (*n* = 47) guided meditation. The animation group was given an animated meditation intervention, and the audio group was given an audio meditation intervention, both interventions were performed 3 times a week and last for 8 weeks. Results: (1) Animation-guided meditation significantly increased participants’ mindfulness and creativity levels; Significantly reduced their cognitive load compared to audio-guided meditation. (2) Mindfulness has a significant direct effect on creativity, and significant indirect effects on creativity; Flow and PA act as the mediating variable. Conclusion: (1) Mindfulness, flow, and PA all helped to improve the subjects’ work creativity. In addition to the direct positive impact of mindfulness on creativity, mindfulness can also have an indirect positive impact on creativity through flow and PA. (2) Compared with audio, animation can significantly reduce cognitive load and help improve users’ cognitive ability, which is more suitable for the guidance materials of mindfulness meditation to enhance the effect of meditation.

## Introduction

### Meditation and Creativity

Creativity is indispensable to the development of human civilization and plays a vital role in the field of human cultural life. It is creativity that has led to so many new inventions and discoveries in human society (Kaufman, 2018). With the trend of intelligent technology, information network and economic globalization, science and technology are changing with each passing day. In this era of encouraging innovation, people are eager to find a simple way to enhance creativity (Sternberg et al., 2020). Creativity includes the ability to create, to generate new and unknown ideas or products, to invent or express imagination and intelligence, and to inspire the power of imagination and invention (El-Murad and West, 2004). The definition of creativity varies, but most scholars agree that creativity is related to three key thinking abilities: divergent thinking, distant association and insight (Dietrich and Kanso, 2010; Sawyer, 2011; Zhang et al., 2020).

Meditation originated in Buddhism, and as meditation became more popular, scholars began to study its physiological and behavioral effects, including its impact on human creativity (Sarath, 2006). Meditation is a combination of emotional and attentional techniques for relaxation, and mindfulness meditation is the most popular form of meditation (Charoensukmongkol and Puyod, 2020). Mindfulness is defined as a nonjudgmental awareness that trains the meditator to focus on the present moment (Agnoli et al., 2018). Mindfulness is also known as a flexible state of mind in which people are actively engaged in the present moment, are more aware of new things, and are more sensitive to external stimuli and environmental changes (Charoensukmongkol and Pandey, 2021). Attention and awareness are key components of mindfulness (Charoensukmongkol, 2019a). Through attention training, people can control their physical and mental activities and get rid of negative emotions (Glicksohn and Ben-Soussan, 2020). Meditation training can also improve cognitive functions such as attention and memory (Lebuda et al., 2016b). As mindfulness meditation has evolved, and more people benefit from mindfulness meditation, there is a trend toward mindfulness-based “cognitive behavioral therapy.” This can largely be attributed to the apparent efficacy of mindfulness meditation in emotional management, interpersonal communication and cognitive function (Charoensukmongkol, 2017). Mindfulness meditation has been found to be associated with improved cognitive function, working memory and the ability to flexibly switch ideas, helping to suppress mental wandering (Charoensukmongkol, 2019b).

Many previous studies have found that mindfulness improves creativity. Divergent thinking is a way of thinking that produces many novel ideas. Mindfulness meditation requires individuals to be open to perception and acceptance of any sensory and external stimuli, and this tolerance and acceptance can promote divergent thinking (Henriksen et al., 2020). Henriksen et al. (2020) found a significant positive correlation between the level of mindfulness and the score of creative expression. Research by Richard et al. (2017) explored the relationship between mindfulness and insight, which is an important component of creative thinking. The resolution of Epiphany problems is easily hindered by automatic language conceptualization processes, and mindful meditation involves “unconceptualized consciousness,” whose purpose is to limit an individual’s ability to automatically activate language conceptualization from previous experiences. Therefore, Tan et al. (2021) propose that mindfulness may contribute to the resolution of insight problems. The results showed that the level of mindfulness was positively correlated with the problem solving rate of insight problems, but not with the problem solving rate of non-insight reasoning problems. Furthermore, the positive correlation between mindfulness and insight remained after controlling for positive emotions and mindful manipulation, suggesting that the relationship between mindfulness and insight was not affected by positive emotions. The results also showed that participants in the mindful group performed significantly better on insight questions than the control group, while there was no significant difference between the two groups on non-insight questions. This provides strong support for a positive correlation between mindfulness and creativity. The study of Ostafin and Kassman (2012) mindfulness-creativity showed that the resolution rate of insight problems in the mindfulness training group was significantly higher than that in the sham training group and the control group, but there was no difference between the sham training group and the control group, proving that mindfulness can improve creativity rather than the placebo effect. Therefore, based on the above results, we can predict that there is a positive correlation between mindfulness and creativity, and mindfulness training may help improve creativity.

There are some common features between mindfulness and flow in that both emphasize focus on the present moment (Wright et al., 2006). Norsworthy et al. (2017) found that flow requires unconscious attention to specific tasks in the present moment. Some scholars have suggested that being present is an effective strategy for achieving flow (Rogatko, 2009; Weinstein et al., 2009; Landhäußer and Keller, 2012). Mindfulness helps people maintain awareness of the present moment, and thus may be the basis of flow (Kaufman et al., 2009; Aherne et al., 2011; Briegel-Jones et al., 2013). Flow and mindfulness are both important concepts in positive psychology that have been shown to play a positive role in stimulating and promoting creativity (Schutte and Malouff, 2020). Csikszentmihalyi first linked flow experiences to creativity in 1997, arguing that flow is an important precursor to high levels of creativity and innovation (Csikszentmihalyi, 1997). Flow is a peak experience of high immersion in the task at hand, and it is easier to experience flow when the challenge of the task matches the individual’s skill set (Csikszentmihalyi and Rathunde, 1993). Creative thinking processes such as insight often occur in the process of reorganizing problem situational information, which often requires remote connection of thinking information, and all these processes require higher attention of individuals (Kounios and Beeman, 2014). Flow is a state of ecstasy when the attention is highly focused on the task at hand. In flow state, creativity is more easily stimulated (Cseh, 2016). When consciousness and spirit are highly focused, the phenomenon of insight is more likely to occur, thus stimulating and promoting creativity (Yang et al., 2019).

Both mindfulness and flow have been proven to have strong emotional regulation effects (Liu et al., 2021a), significantly enhancing positive affect (PA) and reducing negative affect (NA; Liu et al., 2021b,c). Since the 1990s, psychological researchers have conducted numerous studies on the relationship between emotional states and creativity. According to the emotion expansibility and construction theory (Van Cappellen et al., 2021), PA will expand the thinking space and cognitive scope of individuals in a short time, enhance the flexibility of individual thinking, and facilitate the improvement of creativity (West and Fredrickson, 2020). On the contrary, NA is not conducive to the improvement of individual creativity, because NA will reduce the individual’s cognitive range and thinking space (Liu et al., 2020). Baron and Tang (2011) investigated the relationship between emotion and creativity using emotional heuristics and conceptual categorization task execution. The results showed that the experiment-induced active association made the subjects generate more concept categories in the concept classification task, and the active association also made the subjects generate more unusual associations for neutral words. Benedek et al. (2014) also found that PA can promote creative problem solving. Chen and Hou (2016) studied the relationship between creativity and emotion and found that creativity was negatively correlated with NA. Research shows that negative emotions, like depression and anxiety, can get in the way of creative problem solving. He suggests that short-term mind–body conditioning training can improve the cognitive neural mechanisms of creativity. NA reduces the possibility of creative problem solving by limiting people’s attention and ossifying their responses. Davis et al. (2017) also suggested that excessive anxiety would hinder the generation of new and innovative ideas.

To sum up, both mindfulness and flow have positive effects on creativity. Mindfulness may be the basis for the flow experience. Both mindfulness and flow can significantly improve PA. And PA can increase cognitive flexibility and improve the ability of long-distance association, thus promoting creativity. Therefore, flow and PA may play a certain mediating effect on the influence of mindfulness on creativity.

### Animation and Cognitive Load

The widespread use of computers has made multimedia interventions in learning more and more common. Animation is favored by educators, and learners can also refer to the corresponding animation to enhance the learning effect. Animation is a dynamic image, which will change its properties over time to express the functional meaning, use method, state or state transformation of the image itself (Yang et al., 2018). Animation as a way of presentation in teaching has three characteristics: (1) attracts learners’ attention and stimulate learning motivation; (2) describes events that have motion or trajectory; (3) explains complex concepts or phenomena (Aysolmaz and Reijers, 2021). According to congruence principle, the external representation of the animation needs to be highly consistent with the internal information to be expressed, so that the animation can be more easily interpreted and comprehended (Tversky et al., 2002).

Cognitive Load refers to the sum of psychological resources necessary for information processing. Cognitive load theory holds that human cognitive resources (mainly reflected in working memory capacity) are limited. Limited working memory capacity makes it difficult for people to process multiple kinds of information at the same time (Sweller, 1994). If they engage in several activities at the same time, there will be the problem of resource allocation, which follows the principle of “more here and less here, but the total amount remains unchanged.” When a material contains multiple elements interacting with each other, it will increase the cognitive load of learners. If the total amount of Cognitive resources needed in the process of problem solving or learning exceeds the total amount of individual Cognitive resources, it will cause Cognitive overload and affect the effect of learning or problem solving (Sweller, 2011).

According to the different sources of cognitive load, Sweller divided cognitive load into intrinsic cognitive load (ICL), extraneous cognitive load (ECL) and germane cognitive load (GCL; Sweller et al., 1998). (1) ICL refers to the complexity of learning materials – how many elements are composed and how these elements affect each other. When there are many components and their interactions are complex, the ICL is high. ICL is related to the degree of correlation between the complexity of learning materials and the level of learners’ previous knowledge and experience. If the learning materials are complex and the learners lack previous knowledge and experience in this field, they need to process multiple elements at the same time, thus increasing the working memory burden and producing a high ICL. If a learner has more prior knowledge and experience in a certain field, he or she will have less ICL when learning the same material than a learner with less prior knowledge and experience, because the complexity of the learning material is reduced for them (Szulewski et al., 2020). (2) ECL depends on the way information is designed, the way materials are organized and the way they are presented. It is caused by psychological activities that do not directly contribute to learning in the process of learning, which is also known as invalid cognitive load and has a hindering effect on learning. When the information is not well designed, learners must engage in irrelevant or ineffective cognitive processing, which will result in ECL. For example, if the teaching materials include text and diagram, but the text and diagram are not completely consistent in content, it will cause ECL to learners, thus affecting the learning effect. When the information is well designed, the ECL is minimized (Permana et al., 2019). (3) GCL, also known as effective cognitive load, occurs when learners do not use up all cognitive resources in learning a task, and then learners can use the remaining cognitive resources in processing directly related to learning. GCL enables learners to add more advanced conscious cognitive processing (such as reorganization, abstraction, comparison, reasoning, etc.) into the processing activities of working memory. Such processing also increases cognitive load, but rather than impeding learning, it promotes it. The three components of cognitive load can be added, and the allocation of cognitive resources follows the principle of more of this and less of that, with the total amount unchanged. If the total amount of the three cognitive loads does not exceed the total amount of working memory resources, learning can be successfully completed; otherwise, it is not conducive to learning (Albus et al., 2021).

Compared to text, static graphics and pure sound, animation have the following advantages in reducing cognitive load.

1. Reduce ECL. Since the ECL is mainly caused by the organization and presentation of learning materials, optimizing the presentation of learning materials is an important way to reduce the ECL of learners. The study showed that the animation samples presented by the integration of audio-visual and audio-visual methods in multimedia teaching could reduce the cognitive load of learners, and the effect was better than that presented by visual or auditory methods alone, and the learners’ transfer performance was higher. Some scholars suggested that watching and listening channels use different types of working memory through different information processing methods. Therefore, animation presents information in both audio and visual way, which increases the capacity of the total working memory and reduces the cognitive load of using a single channel to present information, thus leading to better learning effect (Mousavi et al., 1995).
2. Increase GCL. Researchers treated motivation as a variable by using real learning materials in real Settings (De Backer et al., 2022). They suggest that motivation may be a key factor in instructional design to promote learners’ learning and improve their academic performance. Recently, many researchers found that learners’ self-interpretation technique is an effective method to increase GCL. Studies have shown that animations can attract users’ attention and motivate them to learn (van Brussel et al., 2021). Researchers proposed that inductive learning strategies and meta-cognitive monitoring cues can increase learners’ GCL and improve their transfer performance. Meta-cognitive monitoring is a kind of control of learners’ learning process, which enables learners to focus more attention on learning content and carry out learning more deeply. Although meta-cognitive monitoring activities also consume cognitive resources and increase cognitive load, these cognitive resources are used in activities directly related to learning, which can improve the learning effect. Therefore, the increase of GCL can help reduce the overall cognitive load (De Backer et al., 2022).

### Research Purpose, Model and Hypotheses

Meditation practice requires the practitioner to master certain methods and techniques, and it is difficult for beginners without training experience to enter the state of mindfulness. The systematic study of mindfulness meditation usually takes weeks or even months. How to find a simple and easy way to let inexperienced beginners, busy professionals in a relatively short time to master the method of meditation quickly into the meditation state. Animation is often used as the carrier of training learning and description materials, with a better guiding effect. Many studies have shown that animation can improve people’s comprehension and cognitive ability of learning material. Animation is used to make guiding content, and sound and pictures are used to make the subjects relax, so that the subjects can consciously pay attention to their own attention, so as to guide them into a relaxed state. The holistic mind–body conditioning training does not require efforts to control the mind and emphasizes the harmony and balance between the body and the mind, leading the subjects into a meditative state. Therefore, this study uses animation as the guide material carrier of meditation, aiming to explore: (1) the effect of animation-guided meditation on enhancing creativity; (2) to explore the role of flow and affect in the influence of mindfulness on creativity.

**Figure 1:**
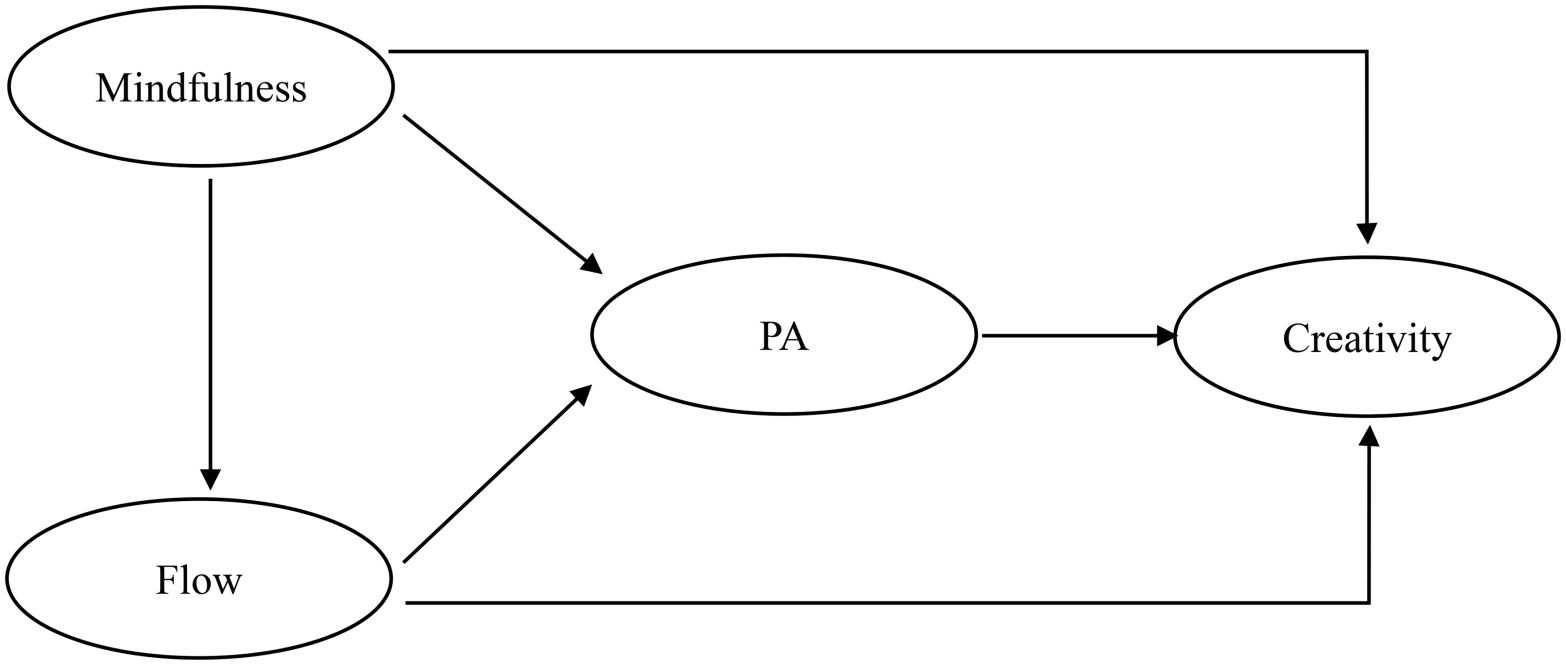
Research model.

Based on the above literature review, we can draw the model framework (Figure 1) of this study and put forward the research hypotheses.

*Hypothesis 1*: Compared with other forms of meditation learning materials, animation can significantly reduce subjects’ cognitive load and significantly improve their meditation effect (mindfulness, flow, PA and creativity).

*Hypothesis 2*: Mindfulness has a direct positive effect on creativity, and has indirect positive effects on creativity through flow and PA.

## Materials and Methods

### Participants

The participants of this study are employees working in the creative industry (Digital media, film post-production, animation, game development and other companies) in China. We advertised recruitment through the internal network of a creative industrial park. One hundred subjects were recruited, and the final 95 eligible participants were divided into two groups: animation (*n* = 48) and audio (*n* = 47) guided meditation, and strictly match the proportion of the gender and education levels, so there was no significant difference in demographic factors between the two groups (Table 1). The informed consent of each participant was obtained.

**Table 1:** Demographic characteristics of participants.

Characteristic		Total	Animation	Audio
Age (SD)		34.57 (8.13)	33.72 (8.45)	35.41(7.96)
Gender	Male (%)	71 (75%)	36 (75%)	35 (74%)
	Female (%)	24 (25%)	12 (25%)	12 (26%)
Education	Associate degree (%)	31 (33%)	16 (33%)	15 (32%)
	Bachelor degree (%)	46 (48%)	23 (48%)	23 (49%)
	Master degree (%)	18 (19%)	9 (19%)	9 (19%)

### Measures

1. Work creativity scale (WCS). These tests adopt the form of subjective self-evaluation, objective score, easy to operate, but also greatly save measurement time. WCS was originally created by Zhou and George in 2001 (Zhou and George, 2001). The scale consists of 13 items and is graded on a 5-point scale (1 = Strongly disagree and 5 = totally agree). The higher the score, the more obvious the creativity tendency of the individual is. Since this study focuses on the creativity of employees in creative industries, WCS is used as a tool to measure creativity. The Chinese version of WCS was adapted by Xu et al. which has good validity (Xu et al., 2014). In this study, Cronbach’s alpha was 0.87.
2. Mindful Attention Awareness Scale (MAAS), which is used to measure the overall tendency of individuals to pay Attention to and become aware of the present experience in daily life. The scale has 15 items and is scored on a 6-point scale (1 = always, 6 = always not). Individuals were asked to rate how often they were in states such as autopilot thoughts or occupied thoughts. The MAAS scale developed by Brown and Ryan has a small number of questions, simple operation and highlights the important component of mindfulness, “attention-awareness,” which is one of the most commonly used tools in the measurement of mindfulness (Brown and Ryan, 2003). The Chinese version of MAAS was adapted by Deng et al. which has good validity (Deng et al., 2012). In this study, Cronbach’s alpha was 0.93.
3. The Short Dispositional Flow Scale 2 (SDFS-2). This nine item self-report measure is an abbreviated version of the long DFS-2 (Jackson et al., 2008). It is purported to include 9 dimensions and 9 items (one item for one dimensions): challenge-skill balance, action-awareness merging, clear goals, unambiguous feedback, concentration on task, sense of control, loss of self-consciousness, time transformation, and autotelic experience. Items are rated on a 5-point Likert scale, ranging from 1 (never) to 5 (always), to measure the frequency of flow characteristics experienced. By summing the responses of the items, an overall dispositional flow score is generated, which may range from 9 to 45. Higher scores indicate higher levels of dispositional flow. Preliminary studies have shown that the short scale well represents the previously validated longer version, indicating that it is an appropriate and reliable empirical measure of dispositional flow (Jackson et al., 2008). The Chinese version of SDFS-2 was adapted by Liu et al. which has good validity (Liu et al., 2012). In this study, Cronbach’s alpha was 0.89.
4. The Positive and Negative Affect scale (PANAS), developed by Watson et al. (1988), is used to measure participants’ positive and negative emotional experiences. PANAS contains two dimensions: positive emotional experiences and negative emotional experiences. Each dimension has 10 items, for a total of total 20 items. Participants answer using a five-point Likert scale, whereby 1 means “none at all,” and 5 means “all the time.” Each subscale is individually scored, and the two subscales are summed to derive a total score. Previous studies have shown that PANAS has good validity (Horwood and Anglim, 2019). The Chinese version of PANAS, adapted by Sheldon et al. has good validity (Sheldon et al., 2004), has the same two-factor structure. In the current study, the Cronbach’s alpha of each sub dimension: (1) PA (0.91); (2) NA (0.92).
5. Brunken classified various methods for assessing cognitive load in two dimensions: objective reality and causality. Objectivity is divided into two categories: based on subjective, self-reported data or based on objective observations of behavior, performance, or psychophysical states; Causality also falls into two categories: whether the observed phenomenon contributes directly or indirectly to cognitive load. Each method has its own advantages and disadvantages, such as physiological measurement is relatively straightforward, but at present it requires expensive equipment. Therefore, self-report method is still widely used at present. This study mainly used direct measures in the subjective dimension: subjects’ self-reported stress level and material difficulty, and indirect measures in the objective dimension: measures of meditation effects (mindfulness, flow, affect and creativity; Brunken et al., 2003). In this study, Cronbach’s alpha was 0.90.

### Experimental Procedure

We advertised on the intranet of a creative industrial park and the employees who were interested and eligible to participate in our study provided their registration information. The recruiting advertisement said that there was a free mindfulness meditation training program to help regulate mood and boost creativity. Interested employees can sign up. Inclusion criteria were: (1) adults aged 18 years or older; (2) able to speak and read in Chinese sufficiently to complete the questionnaire; (3) have at least 1 year working experience in creative industry. Exclusion criteria were: (1) self-reported depression, anxiety, bipolar disorder, substance abuse or suicide; (2) prior experience with mindfulness meditation. Three materials for guiding meditation were used in this experiment: text, audio and animation. The information content contained in the three media materials is the same, and they all explain and guide how to conduct mindfulness meditation. The audio files used in the experiment included a human reading instructions and soothing music in the background. The audio file and the animation file were exactly the same in the sound part, the only difference was that the animation file had pictures, while the audio file had no picture. The content of the text file is the corresponding text of the human reading instructions in the audio and animation. Text files were used to assess cognitive load during pre-test to establish a baseline for cognitive load.

**Figure 2:**
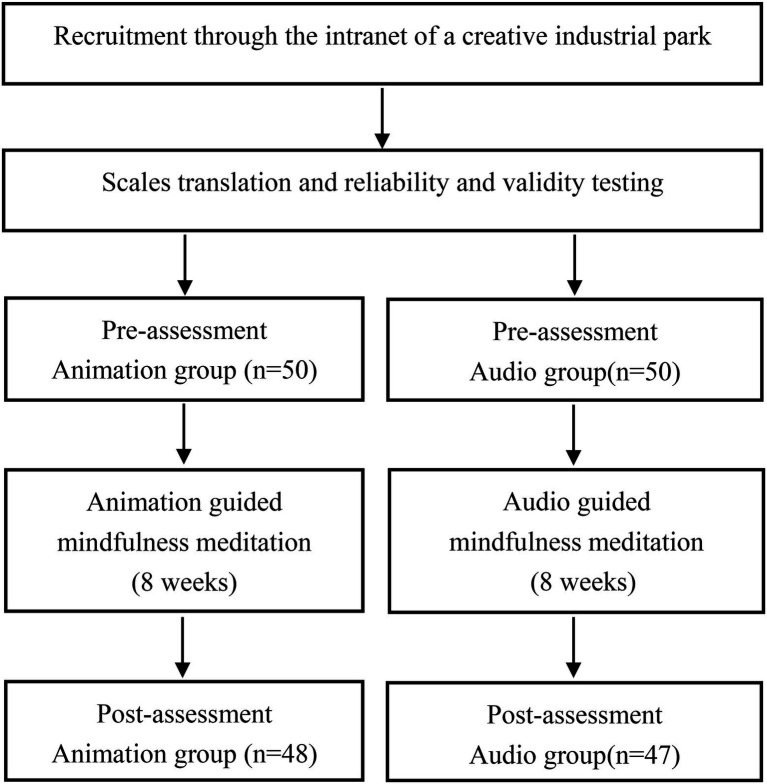
Procedure flow chart.

Participants complete an online consent form through a secure online survey platform, provide media materials, demographic information and the following questionnaires (pre-test): (1) MAAS; (2) PANAS; (3) DFS-2; (4) WSC; (5) SLS. It took approximately 20 min to answer the questionnaire. In order to establish a baseline for cognitive load, participants in both groups were assessed during pretest using the instruction manual to guide mindfulness meditation. Animation group received animation meditation intervention 3 times a week for 8 weeks; the audio group received the same frequency and duration of audio meditation intervention. Both the two groups were invited to a 20-min zoom webinar which provided an overview of how to use the animated and audio meditations. All participants began the 8-week study on the Monday following the zoom webinar. At the end of the intervention period (week 8), participants completed the same questionnaire again (Figure 2).

This study followed an online funnel reporting procedure. To recruit subjects who were oblivious to the experimental conditions and naively used animated meditations, the funnel report helped to obtain a homogeneous sample in both groups. Participants were given the opportunity to record any number of questionnaires anonymously and could withdraw at any stage.

### Data Analysis

SPSS 22 software was used for ANOVA. Partial least squares (PLS) 3 is used to assess the reliability and discriminant validity, and verify the path relationship of the research model. The significance level was set to 0.05.

## Results

### Analysis of Variance

**Figure 3:**
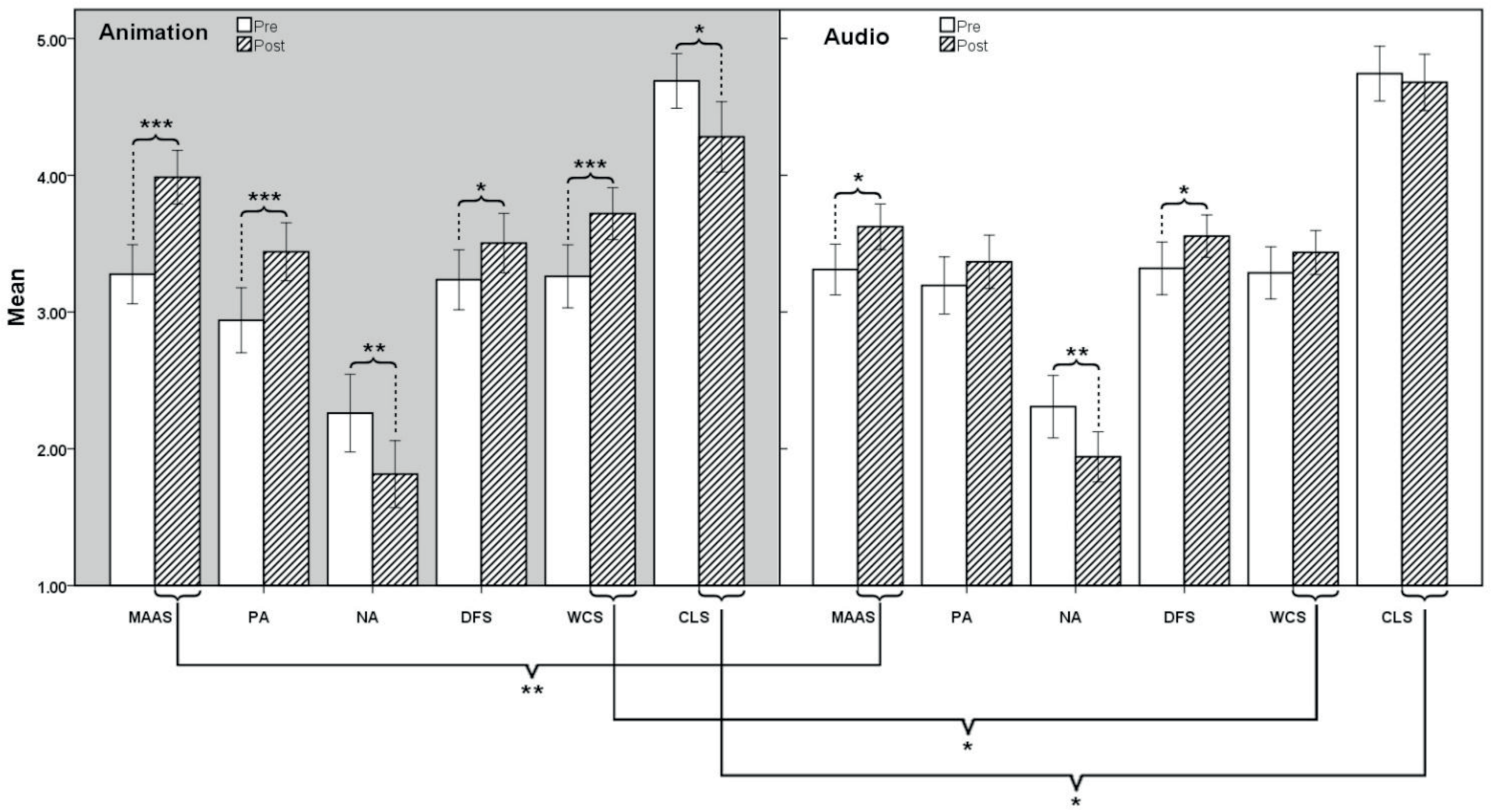
Comparison of 6 measures between animation and audio group. Only significant differences are marked with ^*^p < 0.05; ^**^p < 0.01; ^***^p < 0.001.

In order to compare the guidance effect of different media forms (animation and audio) on mindfulness meditation. This study conducted six 2 (Time: Pre, Post) × 2 (Group: Audio, Animation) ANOVA with repeated measures on mindfulness (MAAS), positive affect (PA), negative affect (NA), flow (DFS-2), work creativity (WCS), and cognitive load (CLS). In order to test for the effects of group type differences in how different guidance media influence the effect of mindfulness meditation, the two factors (gender and education) were controlled and excluded from the ANOVA. The values of *p* Box’s test and Mauchly’s test were all greater than 0.05, showed that the observed covariance matrices of the dependent variables are equal across groups, indicating that these data were suitable for ANOVA. The descriptive statistics are presented in Table 2, and the results of the ANOVA are presented in Table 3 and Figure 3.

**Table 2:** Descriptive statistics.

Group	Measure	Mean (SD)	
		Pre	Post
Animation	MAAS	3.277(0.745)	3.986(0.675)
	PA	2.940(0.817)	3.440(0.730)
	NA	2.261(0.979)	1.815(0.843)
	DFS-2	3.236(0.754)	3.504(0.749)
	WCS	3.261(0.794)	3.719(0.654)
	CLS	4.689(0.687)	4.281(0.885)
Audio	MAAS	3.311(0.630)	3.624(0.568)
	PA	3.195(0.713)	3.368(0.663)
	NA	2.308(0.777)	1.941(0.623)
	DFS-2	3.320(0.656)	3.555(0.528)
	WCS	3.287(0.649)	3.436(0.548)
	CLS	4.743(0.682)	4.679(0.701)

**Table 3:** ANOVA results.

Measure	Variable	*F*	*p*	*η*2
MAAS	Time^***^	30.875	< 0.001	0.249
	Group	2.740	0.101	0.029
	Time ×Group^*^	4.636	0.034	0.047
PA	Time^***^	17.434	< 0.001	0.158
	Group	0.511	0.476	0.005
	Time ×Group^*^	4.119	0.045	0.042
NA	Time^***^	23.590	< 0.001	0.202
	Group	0.357	0.552	0.004
	Time ×Group	0.227	0.635	0.002
DFS-2	Time^**^	10.442	0.002	0.101
	Group	0.338	0.563	0.004
	Time ×Group	0.044	0.834	<0.001
WCS	Time^***^	17.724	< 0.001	0.160
	Group	1.229	0.270	0.013
	Time ×Group^*^	4.601	0.035	0.047
CLS	Time^*^	5.170	0.025	0.053
	Group^*^	4.072	0.046	0.042
	Time ×Group	2.754	0.100	0.029

^*^p < 0.05; ^**^p < 0.01; ^***^p < 0.001.

According to the results of ANOVA, both animation and audio guided meditation can significantly improve participants’ mindfulness and flow level, and reduce NA. In addition, the animation guided meditation significantly increased the subjects’ level of work creativity and PA, and significantly reduced the cognitive load on the meditation guided material. In the comparison between the two groups, the animation-guided meditation significantly increased participants’ mindfulness and creativity levels and significantly reduced their cognitive load compared to audio-guided meditation. There was no significant difference in affect performance between the two groups, but there was a significant interaction effect (Time × Group) of PA. Hypothesis 1 is basically supported.

### Partial Least Squares

To assess the reliability, the composite reliability (CR) and the average variance extracted (AVE) are calculated. Table 4 shows that the CRs of the constructs range from 0.946 to 0.973, which are all above the 0.70 recommended level. The AVEs of the items range from 0.541 to 0.783, which are all above the 0.50 recommended level.

**Table 4:** Reliability and Convergent validity of constructs.

Construct	Reliability		Convergent validity
	Cronbach’s Alpha	CR	AVE
MAAS	0.940	0.946	0.541
PA	0.953	0.960	0.706
NA	0.970	0.973	0.783
DFS-2	0.941	0.951	0.683
WCS	0.968	0.972	0.727

To assess the discriminant validity, Table 5 shows that the squared root of the AVE of each construct is larger than its correlations with other constructs. Therefore, the reliability and discriminant validity are supported by all of the constructs in this study.

**Table 5:** Inter-construct correlations and discriminant validity.

	DFS-2	MAAS	NA	PA	WCS
DFS-2	0.826				
MAAS	0.528	0.736			
NA	−0.112	−0.115	0.885		
PA	0.712	0.634	−0.156	0.840	
WCS	0.694	0.727	−0.191	0.650	0.853

Table 6 summarizes the theoretical effect size results for *R*^2^ and *f*^2^. *R*^2^ is the index of coefficient of determination of the endogenous variables. *R*^2^ greater than 0.67 indicates strong explanatory power; *R*^2^ between 0.33 and 0.67 indicates moderate explanatory power; *R*^2^ less than 0.19 indicates weak explanatory power. And *f*^2^ is the influence index of exogenous variables on endogenous variables. *f*^2^ greater than 0.35 indicates high impact effect; *f*^2^ between 0.15 and 0.35 indicates moderate effect; *f*^2^ less than 0.15 indicates low impact effect. According to the results in Table 6, the structural model of this study has an acceptable evaluation validity.

**Table 6:** Theoretical effect sizes for *R*^2^ and *f*^2^.

	*R* ^2^	*f* ^2^				
		DFS-2	MAAS	NA	PA	WCS
DFS-2	0.508			0.078	1.048	0.663
MAAS		1.031		0.131	0.476	0.697
NA	0.113					0.147
PA	0.747					0.194
WCS	0.836					

The specific *β* value and corresponding *t*-value and significance of each path are shown in Table 7, the results was calculated using the bootstrapping method and repeated sampling 5,000 times.

**Table 7:** Path coefficients of research framework.

	*β*	SD	Confidence interval		Significance	
			2.50%	97.50%	*t*	*p*
Direct effects						
DFS-2— > PA	0.271	0.120	0.098	0.562	2.256	0.024
DFS-2— > WCS	0.627	0.074	0.526	0.819	9.046	<0.001
MAAS— > DFS	0.470	0.153	0.167	0.731	3.079	0.002
MAAS— > PA	0.547	0.129	0.235	0.721	4.239	<0.001
MAAS— > WCS	0.714	0.120	0.450	0.900	5.946	<0.001
PA — > WCS	0.311	0.116	0.017	0.467	2.690	0.007
**Indirect effects**						
MAAS— > PA— > WCS	0.478	0.070	0.360	0.618	6.796	<0.001
MAAS— > DFS-2— > WCS	0.523	0.081	0.359	0.656	6.466	<0.001
DFS-2— > PA— > WCS	0.229	0.101	0.010	0.389	2.273	0.023
MAAS— > DFS-2— > PA— > WCS	0.163	0.069	0.007	0.277	2.373	0.018
**Total effects**						
MAAS— > WCS	0.908	0.096	0.683	1.042	9.461	<0.001
DFS-2— > WCS	0.734	0.112	0.485	0.896	6.556	<0.001

**Figure 4:**
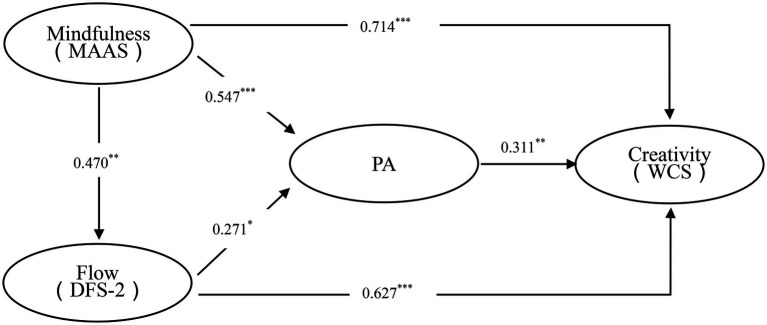
Research framework. ^*^p < 0.05, ^**^p < 0.01, and ^***^p < 0.001.

From the model of research framework in Figure 4, we can see that mindfulness has a significant direct effect on creativity, and significant indirect effects on creativity in which flow and PA acts as the mediating variable and has a significant partial mediating effect, so hypothesis 2 is also supported.

## Discussion

### Mindfulness and Creativity

The study found a positive correlation between levels of mindfulness and creativity. This is consistent with our research hypothesis. Previous studies have found that the higher the level of mindfulness, the better the performance of creativity, and mindfulness training has a significant promotion effect on creativity (Byrne and Thatchenkery, 2019; Ngo et al., 2020). Lebuda et al. (2016a) conducted a meta-analysis of the relationship between mindfulness and creativity, and the results also supported the positive correlation between mindfulness and creativity. Focusing on the present, the core element of mindfulness, allows subjects to remain aware for a long time and promotes mental activity, thus helping to stimulate creativity (Henriksen et al., 2020). Non-judgmental acceptance, another core element of mindfulness, enables employees to accept multiple information and break fixed thinking, making it easier for them to clash and brainstorm ideas, which is conducive to employees’ coming up with innovative ideas. Experimental studies of mindfulness in business Settings have also shown that increasing mindfulness levels can improve employee creativity and avoid mental exhaustion (Lomas et al., 2019). Mindfulness helps trainers to free their attention from the internals of problems, view them in a more inclusive and accepting way, and promote their ability to shift perspectives (Lebuda et al., 2016b). Through this process, practitioners separate their thinking from patterns that hinder the creative process and are no longer bound by habitual responses, thus preventing individuals from falling into stereotyped thinking (Lippelt et al., 2014). Stereotyped ways of thinking can prevent individuals from looking at problems from new perspectives, thus hindering creativity. Mindfulness, a form of introspection, requires people to bring their attention to the present moment. This reassessment leads to a reorganization of the problem situation, which also leads to a deeper understanding of the current problem (Wiranti and Inten, 2012). In addition, mindfulness improves the ability to regulate emotions, which is considered a potential contributor to creativity (Henriksen et al., 2020). Among the cognitive and affective factors of mindfulness, perspective-shifting ability and emotion regulation ability are important factors promoting creativity. Mindfulness implies an unjudgmental awareness of the present and requires a conscious break from the automatic mental processes of cognitive evaluation (e.g., good and bad, useful and useless; Shen et al., 2021). In order to gain insight into an object and put forward new ideas and solutions, it is necessary to examine the object objectively and calmly and avoid relying on previous empirical assumptions (Karwowski, 2021). Breaking previous assumptions and building new knowledge means a certain degree of creativity. Therefore, individuals with higher level of mindfulness have higher awareness and concentration, can break through stereotyped thinking and avoid adopting immutable methods to solve problems. At the same time, they can accurately judge and calmly analyze things, and eventually come up with new ideas, showing high creativity (Bartlett et al., 2019).

### Flow and Creativity

The results of the correlation analysis between flow and creativity in this study show that creativity has a significant positive correlation with flow. And flow positively predicts creativity. It shows that if subjects can have clear goals and devote themselves to an activity they are interested in, they can only pay attention to the development and changes at this moment, so that they can exert greater creative potential (Schutte and Malouff, 2020). The findings are consistent with Csikszentmihalyi’s conclusion that fluid experiences can be important forerunners of high levels of creativity and innovation. In the process of creativity, emotions and flow will be involved. Without positive emotional support, creativity can hardly be maintained (Csikszentmihalyi, 2015).

Mindfulness promotes the flow at the beginning, because both mindfulness and flow are focused on awareness of the present moment (Wright et al., 2006). But when the level of mindfulness is high, mindfulness will have an inhibitory effect on flow, because if go further on the basis of awareness, mindfulness requires maintaining awareness of external stimuli and inner activities, and flow requires further ecstasy, complete absorption and immersion in the task, so at this time the two have different development paths (Sheldon et al., 2015). The results of this study found that both the animation group and the audio group significantly improved mindfulness and flow. The animation group has a significant improvement in mindfulness than the audio group, but there is no significant difference in flow, which also confirms this view.

### Affect and Creativity

This study found a significant positive correlation between PA and creativity. This is consistent with previous theories and research conclusions. From the perspective of psychology, PA can create a positive psychological state and open mind, promote the cognitive flexibility of individuals, expand their cognitive range, improve the speed of information processing, and improve the cognitive fluency and response level (de Rooij and Vromans, 2020). On the other hand, NA will narrow an individual’s attention, confining it to narrow details, reducing their cognitive flexibility and expansibility, and thus inhibiting an individual’s creative potential (He et al., 2020). Dan (2021) found that people with higher PA levels have greater cognitive flexibility, which enables him or her to accept different cognitive elements and connect them to find creative solutions. PA has been found to enhance cognitive flexibility and promote creativity by creating more diverse connections between perception and thought (Hensley, 2020). PA allows people to think flexibly and generate more novel ideas. In general, highly creative individuals are more emotionally stable and have higher energy levels (Tavares, 2016). Some studies have found that PA expands attention span and promotes the formation of distant associations. Some scholars suggested that PA enhances the ability to switch between global attention mode and local attention mode and the ability to choose between different perspectives (Rego et al., 2012). Conversely, NA states (such as anxiety and depression) are associated with attentional deficits and maladjustment of cognitive control mechanisms, and NA often leads to a narrowing of attention span (Leung et al., 2021). Therefore, NA should have the effect of impeding cognitive flexibility and creative problem solving. In addition, from the neurophysiological perspective, PA can promote the secretion of dopamine in the brain, and dopamine released by the anterior cingulate cortex can regulate and improve the cognitive fluency, cognitive reorganization, process focusing and information integration of the brain, thus stimulating a higher level of creativity (Parke et al., 2015). Mooer et al. (2018) reached a similar conclusion through cognitive neural experiments. Their results showed that the visual cortex of the brain can process more information when the subjects are in a positive mood, but when subjects were in a bad mood, their ability to acquire information was significantly limited.

The results showed that emotional regulation partially mediated the relationship between mindfulness and creativity, suggesting that mindfulness not only directly affected creativity, but also indirectly affected creativity through the two dimensions of emotion. When individuals have a high level of mindfulness, they will focus on their current internal experience without judgment and are more likely to generate PA and maintain happiness, thus actively accepting more new things and generating more new ideas (Ovington et al., 2018). Individuals with low level of mindfulness are more difficult to detect their own emotional changes and adjust them. When he or she is in a negative emotional state, his or her creativity is negatively affected (Henriksen et al., 2020).

### Advantages of Animation

Well-designed animation helps mentally visualize a process or program, leading to a reduction in cognitive load (Höffler and Leutner, 2011). Animations provide a more realistic representation of content and explicitly describe dynamic information without the need for users to infer content, thus saving cognitive effort on the psychological construction of animation by users (Tunuguntla et al., 2008). By clearly showing the micro steps required between each important change, the animation adapts to the presentation of a continuous phenomenon, since the user do not need to infer how the phenomenon changes from one step to the next (Tversky et al., 2020). It can be seen from the results that animation is more consistent with the congruence principle of content expression than audio. The animation gives a more complete explanation to the information. Although more elements are added to the animation and the form of expression looks more complicated, but these elements can help explain the concepts and steps of meditation. The degree of complexity may not be the key factors affecting comprehensibility, but rather whether relevant to the message you want to convey (Chen et al., 2021). Animation tells a relatively complete story, explaining the concept and the context of the original information. From the perspective of communication, the interpretation of a particular information may not be independent of context, and the influence of contextual clues may be sensitive (Vigoroso et al., 2020). Therefore, although animation has more information than audio, most of them are closely related to the theme and conform to the principle of consistency, helping users to deepen their understanding of meditation and significantly reducing the cognitive load.

We used cartoon anthropomorphic figures in the animation to demonstrate the movements and essentials of meditation. Because there is both empirical and theoretical evidence to support that animation is an effective way to learn human motion (de Koning et al., 2019). It has been indicated in many studies that animation has great advantages in depicting human-motor skills and many studies used mirror–neuron theory to explain this phenomenon (Brucker et al., 2021). Researchers proposed that humans may have evolved the ability to learn certain types of knowledge effortlessly, such knowledge is called biologically primary knowledge, and human motion belongs to this kind of knowledge (Geary, 2008). Further, Researchers suggested that humans may have evolved specific components of working memory that allow us to naturally acquire visualizations involving human motion (Ayres, 2020). This mirror neuron system and related motion processors are likely to be the key physiological structures that allow humans to participate in learning through observation and imitation (Brucker et al., 2021). In contrast, learning biological secondary knowledge (Geary et al., 2021) such as mechanical systems, or using static graphics to represent human motion, may require more working memory resources, because we do not have the same biological advantages (Chandler, 2009). This phenomenon explains why it is easier to understand animations that depict primary knowledge, than the animations that depict secondary knowledge. Because primary knowledge is relatively easy to obtain, it will not consume a large amount of working memory resources like secondary knowledge, and do not add too much cognitive load (Carreño et al., 2021). Thus, when users watch animations of primary knowledge, they can manage transient visualization more effectively than the animations of secondary knowledge, thereby reducing cognitive load (Ayres et al., 2019).

### Research Limitations and Future Research

This study explores the relationships among mindfulness, flow, affect and creativity, and the advantages of animation-guided meditation, and draws some practical conclusions. However, due to the human resources, material resources and time, it also has the following limitations:

(1) The subjects of this study are all employees of creative industry, who are characterized by high level of wisdom, active thinking and positive and stable emotions. In addition, the sample size is not large enough and the region is not broad enough. Whether the selected subjects are representative and whether the results are universally applicable needs further investigation and verification. (2) This paper adopts the self-reported work creativity scale suitable for evaluating employees’ creativity, which may not fully illustrate the situation of the subjects. (3) Due to the limited research conditions, this study adopted a horizontal study, but there is a possibility of change in mindfulness, emotion and creativity, especially the change of emotion is quite frequent, so this may affect the results of the study. (4) This study is a short-termed intervention, with no follow-up measures. The increase in creativity may be a short-term effect, whether it can be sustained in the long term needs to be further verified by future studies.

Combined with the limitations of this study, the following suggestions are put forward for future research: (1) future research should expand sample size and sample range types as much as possible to improve sample representativeness and universality of research results. (2) In future studies, creativity measurement tools should be further improved to make a more comprehensive evaluation of creativity. (3) Future research can consider using the method of combining horizontal and vertical research to dynamically grasp the relationship between variables, so as to make the research data more convincing and the research results more rigorous. (4) Multiple and longer-term measurements of creativity will be conducted in future studies to verify the sustainability of creativity enhanced by mindfulness meditation.

## Conclusion

Mindfulness, flow, and PA all helped to improve the subjects’ work creativity. In addition to the direct positive impact of mindfulness on creativity, mindfulness can also have an indirect positive impact on creativity through flow and PA, and flow and PA play a partial mediating role. Compared with static text, graphics and audio, animation can significantly reduce cognitive load and help improve users’ cognitive ability, which is more suitable for the guidance and illustration materials of mindfulness meditation to enhance the effect of meditation.

## Data Availability Statement

The raw data supporting the conclusions of this article will be made available by the authors, without undue reservation.

## Ethics Statement

The study was approved by the Ethics Committee of Chang Gung University (IRB No: 201902226B0) and the study protocol was carefully reviewed to ensure compliance with the Ethics guidelines of the Chinese Psychological Society. The patients/participants provided their written informed consent to participate in this study.

## Author Contributions

HC contributed with data collection, findings interpretation, and paper preparation. CL contributed with the experimental design, data collection, statistical analysis, and findings interpretation. C-HC and Y-LC were in charge of data collection, helped to prepare the experimental sites, and assisted with data collection. FZ, KW, D-HH, and C-YL assisted in the data collection and evaluation of the findings, as well as conceiving the project and participating in its interpretation. W-KC was responsible for the study’s conception and design, as well as the interpretation of the findings and paper writing. All authors contributed to the article and approved the submitted version.

## Funding

This research was supported by the Ministry of Science and Technology (MOST), Taiwan, Grant MOST 109-2221-E-182-033-MY3.

## Conflict of Interest

The authors declare that the research was conducted in the absence of any commercial or financial relationships that could be construed as a potential conflict of interest.

## Publisher’s Note

All claims expressed in this article are solely those of the authors and do not necessarily represent those of their affiliated organizations, or those of the publisher, the editors and the reviewers. Any product that may be evaluated in this article, or claim that may be made by its manufacturer, is not guaranteed or endorsed by the publisher.
